# Contribution of *MSMB* promoter region gene polymorphism to early-onset prostate cancer risk in Mexican males

**DOI:** 10.18632/oncotarget.26592

**Published:** 2019-01-22

**Authors:** Silvia Juliana Trujillo-Cáceres, Luisa Torres-Sánchez, Ana I. Burguete-García, Yaneth Citlalli Orbe Orihuela, Ruth Argelia Vázquez-Salas, Esmeralda Álvarez-Topete, Rocío Gómez

**Affiliations:** ^1^ Centro de Investigación en Salud Poblacional, Instituto Nacional de Salud Pública (INSP), Cuernavaca, Morelos, Mexico; ^2^ Centro de Investigación en Enfermedades Infecciosas, INSP, Cuernavaca, Morelos, Mexico; ^3^ Conacyt-Centro de Investigación en Salud Poblacional, INSP, Cuernavaca, Morelos, Mexico; ^4^ Departamento de Toxicología, Cinvestav-IPN, Mexico City, Mexico

**Keywords:** genetic polymorphisms, MSMB, prostate cancer, rs10993994, sexually transmitted diseases

## Abstract

Sexually transmitted infections and its contribution to prostate cancer (PC) development have been relevant in different populations. *MSMB* gene polymorphism (rs10993994) has exhibited an association both with PC as well as the susceptibility to sexually transmitted infections. Hitherto, these conditions have been not studied in Mexico yet, neither if sexually transmitted infections could modify the MSMB and PC association. Herein, socio-demographic features, sexually transmitted infections records, the reproductive backgrounds, and the genetic characterisation were analysed in 322 incident PC cases and 628 population healthy controls from Mexico City. Whole PC, early-onset PC (PC at < 60 years old), late-onset PC (≥ 60 years old), and PC aggressiveness were used to evaluate the genetic variants contribution to PC risk using unconditional logistic regression models. Overall, none associations between the allelic variants of rs10993994 polymorphisms with whole and PC aggressiveness were found. Howbeit, the TT genotype carriers presented the highest susceptibility to develop early-onset PC (OR = 2.66; 95% CI = 1.41, 5.04; *p* = 0.03) than CC+CT carriers, both with codominant and recessive models. Although none association between whole PC and *MSMB* gene polymorphism was found, our results were reinforced by prior studies in European descendent populations, suggesting a contribution between rs10993994 and early-onset PC development.

## INTRODUCTION

Prostate cancer (PC) is the second most common malignant neoplasm in males worldwide and the fifth leading cause of death from cancer in men [1]. Risk factors such as age, diet, sexually transmitted infections (STIs), smoking, and obesity have been associated with PC development [2, 3]. In turn, ethnicity, family history and inherited gene changes also play a meaningful role in this cancer [4, 5].

Matrix metalloproteinase, vitamin D receptor polymorphisms as well as those associated with the androgen’s metabolism have been critical traits in genotype-phenotype association studies [6–8]. Recent studies suggest that gene polymorphisms related to prostatic function may participate in prostatic carcinogenesis and seem to confer susceptibility to STIs [9, 10]. Genome-wide association studies have reported a polymorphic variant located in the promoter region of the β -microseminoprotein (*MSMB*) gene (rs10993994; C>T) associated with higher PC risk [11–14]. T allele has been also associated with the modification of the transcription factor binding sites (i.e., cAMP response element-binding protein) reducing the promoter activity of the *MSMB* gene. The T allele alters the production of MSMB, a prostate secretory protein 94 amino acids (also named PSP94) and one of the most abundant proteins secreted by the prostate [15–17]. This protein, present in blood and seminal plasma, regulates prostate growth inducing apoptosis and also under post-coital conditions of vaginal pH, and low calcium concentration, it presents antimicrobial activity [18–20].

An ethnic variation regarding T allele distribution has been reported [21]; African populations exhibit the highest frequencies (> 0.55) [22], followed by European-derived populations (0.41) [14, 23–26], and Latinos (0.36) [10]. Previous case-control studies have suggest that the T allele carriers present 35% more risk than the C allele bearers [14, 24–28], apparently at the expense of a low degree of aggressiveness (Gleason < 7) [22, 23]. Additionally, in a previous report using the population studied herein, a remarkable association (OR = 2.67; _95%_CI = 1.91 – 3.73) between STIs and PC was found [29].

Mexican population presents a complex genetic architecture where gene frequencies of rs10993994 are still unknown. As far as we know, this genetic variant has not been studied in terms of PC association in Latino populations. In the present study, a genotype-phenotype association between rs10993994 and whole PC, early-onset PC (PC at < 60 years old), late-onset PC (≥ 60 years old), and PC aggressiveness were evaluated. Also, we evaluated whether STIs history modifies this association.

## RESULTS

Cases (67.38 ± 8.09 yr.) and controls (66.59 ± 8.8.80 yr.) age averages were similar (*p* = 0.17) between both groups owing to study design. Of all cases, 37.97% (*n* = 122) were classified as Gleason ≥ 8 and 17.08% (*n* = 55) as early-onset PC.

Descriptive characteristics of study population and its comparison between cases and controls are shown in Table 1. PC frequency was higher in cases born both in central east (OR = 1.92; _95%_CI = 1.33 – 2.76; *p* ≤ 0.01), and eastern (OR = 2.61; _95%_CI = 1.29 – 5.29; *p* ≤ 0.01) regions of Mexico. Rather, the frequency of higher scholarship (i.e., bachelor’s degree or higher; OR = 1.67; _95%_CI = 1.12 – 2.49; *p* = 0.01), family history of PC (at least one first-degree familiar with PC diagnosis; OR 4.25; _95%_CI = 2.32 – 7.79; *p* ≤ 0.01), and the personal history of chronic diseases (OR = 1.95; _95%_CI = 1.48 – 2.57; *p* ≤ 0.01), were also higher in cases than controls.

**Table 1:** Selected characteristics of study population according to cases and controls

Characteristics	Cases *n =* 322	Controls *n =* 628	OR^a^	95% CI		*p* value
Birthplace^b^ Mexico City South Central-West Central-East North East	167 (52.2) 25 (7.8) 29 (9.1) 71 (22.2) 11 (3.4) 17 (5.3)	413 (65.9) 43 (6.9) 49 (7.8) 90 (14.4) 16 (2.5) 16 (2.5)	1.00 1.41 1.44 1.92 1.67 2.61	- 0.83–2.39 0.87–2.36 1.33–2.76 0.76–3.68 1.29–5.29		- 0.20 0.15 < 0.01 0.20 < 0.01
Education Elementary school Middle school High school University	154 (47.8) 48 (14.9) 58 (18.0) 62 (19.3)	282 (44.9) 156 (24.8) 120 (19.1) 70 (11.2)	1.00 0.58 0.92 1.67	- 0.39–0.85 0.63–1.34 1.12–2.49		- < 0.01 0.66 0.01
Marital Status United vs. No united	245 (76.1)	498 (79.3)	0.84	0.61–1.15		0.27
Family history of PC Yes vs. No	33 (10.2)	17 (2.7)	4.25	2.32–7.79		< 0.01
History of Chronic Diseases Yes vs. No	189 (58.9)	264 (42.0)	1.95	1.48–2.57		< 0.01
History of STIs Yes vs. No	79 (24.6)	71 (11.3)	2.55	1.79–3.64		< 0.01
History of Gonorrhea Yes vs. No	62 (19.3)	37 (5.9)	3.80	2.47–5.86		< 0.01
Number of sexual partners ≤ 2 3–6 > 6	74 (23.4) 94 (29.8) 148 (46.8)	207 (33.5) 219 (35.4) 192 (31.1)	1.00 1.22 2.18	- 0.85–1.75 1.55–3.07		- 0.28 < 0.01
Body Mass Index^c^						
Normal Overweight Obesity	170 (27.6) 298 (48.4) 148 (24.0)	324 (26.7) 596 (49.1) 294 (24.2)	1.00 0.96 0.98	- 0.76–1.21 0.75–1.29		- 0.72 0.90
Smoking patterns^d^ No smokers A B	109 (33.9) 184 (57.1) 29 (9.0)	211 (33.6) 371 (59.1) 46 (7.3)	1.00 0.97 1.20	- 0.72–1.29 0.71–2.02		- 0.81 0.49
Physical activity patterns^e^ None A B C	47 (14.6) 80 (24.8) 177 (55.0) 18 (5.6)	63 (10.0) 116 (18.5) 405 (64.5) 44 (7.0)	1.00 0.95 0.59 0.58	- 0.59–1.53 0.39–0.90 0.30–1.13		- 0.84 0.01 0.11

^a^OR adjusted by age at interview.

^b^**Birthplace**: Mexico City (Ref.). South: Campeche, Chiapas, Guerrero, Oaxaca, Quintana Roo and Yucatán. Central-West: Aguascalientes, Colima, Guanajuato, Jalisco and Michoacán. Central-East: Hidalgo, Estado de México, Morelos, Puebla, Querétaro and Tlaxcala. North: Chihuahua, Coahuila, Durango, San Luis Potosí, Zacatecas, Baja California, Baja California Sur, Sinaloa, Sonora, Nayarit, Nuevo León and Tamaulipas. East: Veracruz and Tabasco.

^c^Two years before diagnosis or interview.

^d^**Smoking patterns**: A: males who reported low and constant smoking intensity, behavioral; B: males with an initial period of low smoking intensity, followed by an increase after 30 years old.

^e^**Physical activity patterns**: A: males who had high PA intensity and frequency at 15–18 years old and showed a higher reduction throughout life; B: males who maintained consistently low PA, and C: males who consistently performed more PA.

Regarding the sexual history, 15.82% of the men reported at least one STI event being the most frequent gonorrhoea (10.4%); chancre (1.58%), acquired syphilis (1.05%), genital warts (1.05%) and herpes (0.95%) were the least frequent. It is noteworthy that precedents of any kind of STIs (at least one STI) were higher in cases than in controls (OR = 2.55; _95%_CI = 1.79 – 3.64; *p* ≤ 0.01); particularly the gonorrhoea background (OR = 3.80; _95%_CI = 2.47 – 5.86; *p* ≤ 0.01), was associated with more frequency of PC. The number of sexual partners (≥ 6) through all lifelong was twice associated with the PC frequency (Table 1).

### Genetic statistical analysis

Similar distributions in allele and genotype frequencies were found between cases and controls. Likewise, an important Hardy-Weinberg departure (HWD) related to homozygous excess was found both in cases (*F*_*IS*_ = 0.112; *p* = 0.03) and in controls (*F*_*IS*_ = 0.125; *p* = 0.002). T allele frequency within the controls (Supplementary Table 1) exhibited dissimilar distributions frequencies related to the birthplaces, these differences were more prominent in the Central-West (10%) and South states (4.3%).

About the relationship between T allele with STIs, relevant differences in frequency were found in men with history of herpes. The carriers of two T allele dosages presented a significant seven-fold (2.56 vs 0.36; *p* = 0.02) higher frequency of herpes background in comparison with the CC+CT genotype carriers. Nonetheless, given the scantiness number of individuals with this condition (just four men reported herpes history) it results should be interpreted with caution. The rest of STIs did not show phenotype-genotype interaction.

On the other hand, no association between the risk allele (T) with whole PC (Table 2), and PC aggressiveness (Table 3) were found. Also, we did not observe an interaction between STIs background and the risk allele T (Supplementary Table 2).

**Table 2:** Association between rs10993994 polymorphism in *MSMB* gene and PC by alleles and according to different inheritance models

*MSMB* polymorphism	Cases *n =* 322 (33.9)	Controls^a^ *n =* 628 (66.1)	OR^b^	95% CI	OR^c^	95% CI
Allele C T Codominant CC CT TT Dominant CC CT+TT Recessive CC+CT TT	443 (68.8) 201 (31.2) 160 (49.7) 123 (38.2) 39 (12.1) 160 (49.7) 162 (50.3) 283 (87.9) 39 (12.1)	864 (68.8) 392 (31.2) 314 (50.0) 236 (37.6) 78 (12.4) 314 (50.0) 314 (50.0) 550 (87.6) 78 (12.4)	1.00 1.00 1.00 1.03 0.97 1.00 1.02 1.00 0.96	- 0.81–1.23 - 0.77–1.38 0.63–1.49 - 0.78–1.33 - 0.63–1.44	1.00 1.02 1.00 1.08 0.98 1.00 1.05 1.00 0.95	- 0.82–1.25 - 0.80–1.45 0.63–1.52 - 0.80–1.39 - 0.62–1.44

^a^Hardy-Weinberg equilibrium among controls (Chi-^2^ = 9.78, *p* = 0.002).

^b^Odds ratio adjusted for age at interview.

^c^Model adjusted for age at interview, birthplace and family history of PC.

**Table 3:** Association between rs10993994 polymorphism in *MSMB* gene and PC aggressiveness by alleles and according to different inheritance models

*MSMB* polymorphism	Controls (*n =* 628)	Gleason ≤ 6			Gleason 7			Gleason ≥ 8		
		Cases (*n =* 82)	OR^a^	95% CI	Cases (*n =* 114)	OR^a^	95% CI	Cases (*n =* 116)	OR^a^	95% CI
Alleles C T	864 392	116 48	1.00 0.93	- 0.64–1.34	160 68	1.00 0.94	- 0.68–1.28	156 76	1.00 1.13	- 0.83–1.55
Codominant CC CT TT	314 236 78	40 36 6	1.00 1.29 0.59	- 0.79–2.11 0.24–1.47	63 34 17	1.00 0.74 1.06	- 0.46–1.17 0.58–1.93	54 48 14	1.00 1.38 1.07	- 0.88–2.16 0.55–2.08
Dominant CC CT+TT	314 314	40 42	1.00 1.10	- 0.69–1.77	63 51	1.00 0.82	- 0.54–1.24	54 62	1.00 1.30	- 0.85–1.97
Recessive CC+CT TT	550 78	76 6	1.00 0.53	- 0.22–1.28	97 17	1.00 1.19	- 0.67–2.12	102 14	1.00 0.92	- 0.49–1.73

^a^Models adjusted for age at interview, birthplace and family history of PC.

Under recessive and codominant inheritance models the carriers of the TT genotype, exhibited almost thrice more possibility to present early-onset PC (OR = 2.66; _95%_CI = 1.41, 5.04; *p* = 0.003) than the rest of the genotypes (Table 4). This contribution was preserved even considering variables such as age at interview, birthplace, body mass index (two years before PC diagnosis or interview), and family history of PC.

**Table 4:** Association between rs10993994 polymorphism in *MSMB* gene and PC onset by alleles and according to different inheritance models

*MSMB* polymorphism		Early onset PC			Late onset PC		
	Controls (*n =* 628)	Cases (*n =* 55)	OR^a^	95% CI	Cases (*n =* 267)	OR^a^	95% CI
Alleles C T	864 392	72 38	1.00 1.43	- 0.88–2.33	371 163	1.00 1.00	- 0.79–1.26
Codominant CC CT TT	314 236 78	26 20 9	1.00 0.95 **2.60**	- 0.57–1.58 **1.31–5.14**	134 103 30	1.00 1.13 0.90	- 0.89–1.43 0.63–1.27
Dominant CC CT+TT	314 314	26 29	1.00 1.21	- 0.76–1.93	134 133	1.00 1.07	- 0.86–1.33
Recessive CC+CT TT	550 78	46 9	1.00 **2.66**	- **1.41–5.04**	237 30	1.00 0.85	- 0.61–1.19

^a^Models adjusted for age at interview, birthplace, body mass index two years before interview or PC diagnosis and family history of PC.

### Comparisons with other populations

**Figure 1:**
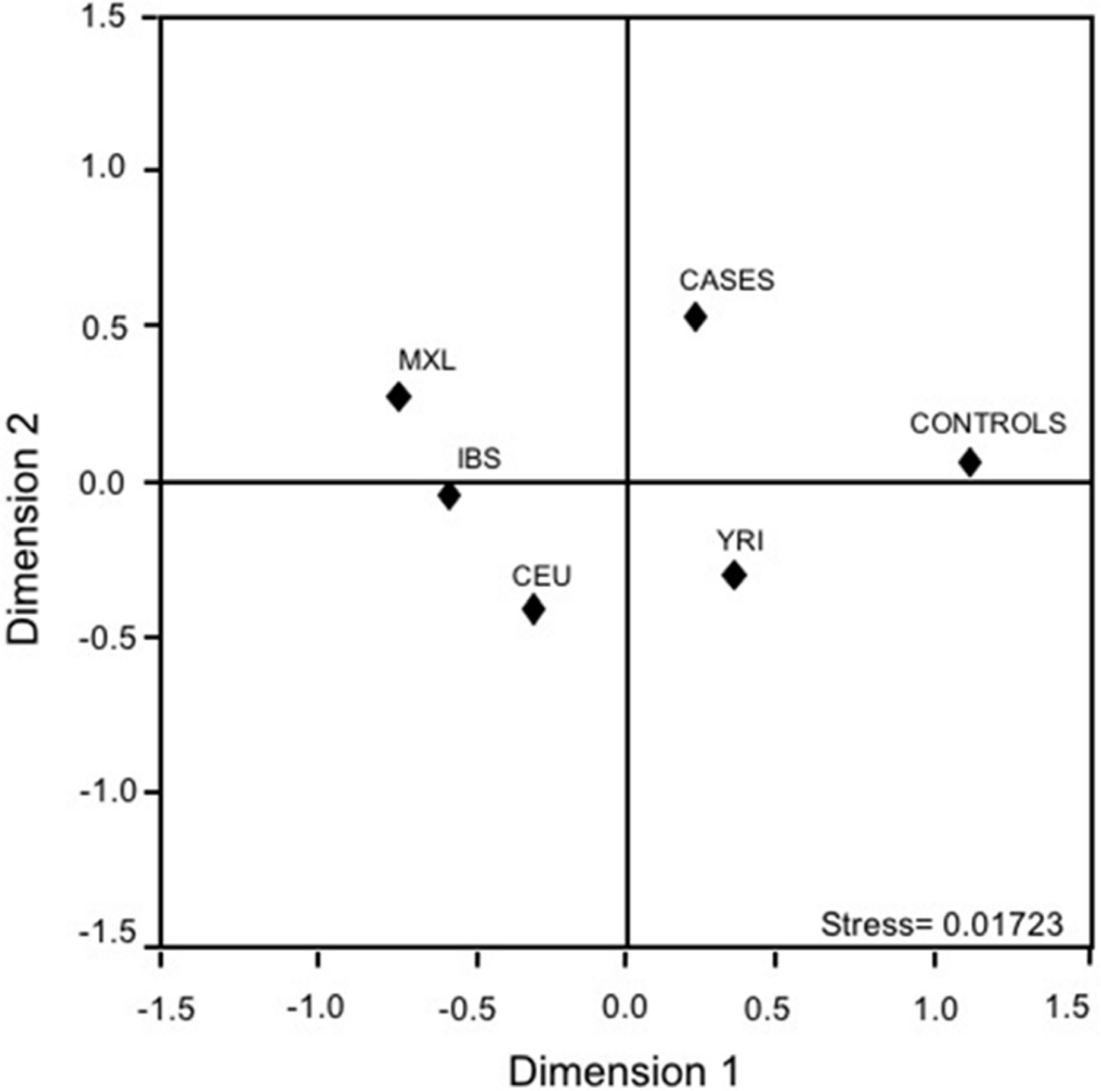
Multidimensional scaling plots of the *MSMB*-rs10993994 polymorphism in cases, controls, and related parental populations CEU: Utah residents with Northern and Western European ancestry from the CEPH collection; IBS: Iberian population in Spain; MXL: Mexican ancestry in Los Angeles, California; YRI: Yoruba in Ibadan, Nigeria.

To compare the rs10993994 gene frequencies with the 1000 genomes data using related descendant populations (i.e., Mexican ancestry in Los Angeles, California (MXL); Utah residents with Northern and Western European ancestry from the The *Centre d’Etude du Polymorphism Humain* collection (CEU); Iberian population in Spain (IBS); Yoruba in Ibadan, Nigeria (YRI)), a complex genetic architecture was depicted. The first dimension separated Cases, Controls, and MXL from the rest of the populations; the second one sets apart Cases, Controls, and YRI from MXL, IBS, and CEU suggesting a possible ancestral relation with the African descendants (Figure 1).

### Y-chromosome analysis

Risk allele of rs10993994 *locus* has been previously related to PC elevated risk, mainly in African derived populations. Thus, a possible relation between the ancestral lineages and early onset-PC was also done. Both the European (37%; G2a, I2b1, and R1b) as the Native American (Q lineage) ancestries were found in similar proportions (38%). The North African and the Middle East lineages (E1b1b, J1, J2a1 x J1a1-bh, J2a1h, J2b, and T; Table 5) were presented in 25% of the early onset-PC. The results were compared with a previous report in the Central Valley of Mexico (CVM) population [32]; the proportions of these two populations (present study early onset-PC *vs* CVM) were analogous.

**Table 5:** Frequency of the haplogroups found in men with early-onset prostate cancer

Geographical origin	Haplogroups	*n*	(%)
Native American	Q	17	34.69
European	G2a I2b1 R1b	18	36.73
North Africa and Middle East	E1b1b J1 J2a1 x J2a1-bh J2a1h J2b T	12	24.50
South of Asia	L	1	2.04
North of Eurasia	N	1	2.04
Total		49	100%

## DISCUSSION

Herein, a susceptibility pattern between the TT genotype and early-onset PC development, aside from the family inheritance (PC family history of first-degree relatives) and the STIs history was found under a recessive heritability model. In discordant to prior reports, none association between rs10993994 polymorphism with the whole PC neither with PC aggressiveness in Mexican males were found. Of note that those studies where the whole PC was associated with the rs10993994 polymorphism have shown a nuanced contribution despite its substantial sample size. Likewise, it is worthwhile to mention that this association was found in European-derived men, where T allele frequency is the most prominent. Thus, it is likely that the lack of association of our study could be related to its modest sample size.

Regarding the number of studies where an association between the risk’s variant with the age of cancer diagnosis was found, it has been scarce hitherto, and most of them only considered dominant and additive heritage models. However, our findings are consistent with some studies in European derived populations where the T allele was associated with early-onset PC (< 55 years old) [13, 33]. Likewise, a study in Scotland population where diagnosis age was not consider found that PC risk was two-fold higher in the TT carriers (OR = 1.87; _95%_IC = 1.26 – 2.77) than in CC+CT genotypes, using identical models those used herein [28]. Worth of note, akin to the results obtained herein, they did not find any association using the dominant model.

In relation to PC aggressiveness, the studies of Fitzgerald *et al.* [23], and Chang *et al.* [22], suggested a possible association between T allele with low-grade PC (Gleason ≤ 6). Nevertheless, our findings did not evidence any association with PC aggressiveness independently of the heritability model used. By contrast to the reports in other worldwide populations where population screening program exists and the low-grade PC frequency ranges between 50 to 84%, in Mexico the observed low-grade PC frequency was low (26%). Hence, it is possible that the association between *MSMB* polymorphism and low-grade PC observed in those population could be a proxy of age at diagnosis. Rather, the results found by Stott-Miller *et al.* [26] supported that the increase of PC among the T allele bearers was independent both the family PC background and personal SITs history, which were consistent with our results. On the other hand, the accordance of the phenotype-genotype association in different ethnicities, as well as the congruousness in gene frequencies across other Hispanic populations reinforced our results. Howbeit, similar gene distributions both in cases than in controls as well as the HWD related to homozygous excess should not pass over. Of note, that this inbreeding was not related with the risk allele. This remarkable inbreeding could be associated with the youth of the Mexican mestizo population, which exhibits at most fifteen generations [34]. Populations such as IBS and even MXL presented *F*_*IS*_ related to the homozygous deficit; CEU and YRI displayed genetic homogeneity in this *locus*.

Previous studies in Mexican Mestizo population [32] have reported little proportions of African derived lineages (7%). These findings were contrasting with the African proportions found in the early-onset PC group (25%). This finding is consistent with prior studies where macro-haplogroup (DE), and sub-haplogroups within E1b1b (i.e., E1b1b1c) have been associated with PC in Japanese and Ashkenazi populations, respectively [35, 36]. Although these lineages have exhibited higher risk than another one in previous findings, our results should be interpreted with caution given that these results represent only a subsample of 49 unrelated individuals.

About the decrease in PSP94 production as a result of the T variant, it has been well documented in previous reports as well as among different ethnicities [10]. PSP94 has been related to prostate tumour growth suppression, presenting less expression in PC advanced stages as those cases refractory to androgens [37]. Given that the association was related to EO-PC, it is likely that the mechanism described before did not stick to our results. Thus, it is likely that the T allele might alter the PSP94 antimicrobial activity, contributing to the prostate chronic inflammatory processes [19]. Nonetheless, this mechanism has not been elucidated yet.

Like any other study, some weaknesses should be considered such as the possibility of misclassifications in relation about STIs history owing to it was evaluated using a questionnaire. This strategy, recurrent in different studies, could skew the precedent history of STIs. Particularly, those STIs transmitted by protozoa (i.e., trichomoniasis), virus (i.e., human papillomavirus), fungi, and yeast (*C. albicans*), could remain silent and present unspecific symptoms [19, 38]. This possible bias could negatively impact in power to detect the interaction between the rs10993994 polymorphism and the STIs (both overall as particularly). Besides, previous reports have suggested that the antimicrobial *MSMB* gene capability could be biological-agent specific. In this setting, a marginal association between the homozygote state of T allele and the genital herpes history was found. This finding, in connection with the sample size, did not allow us to detect a possible interaction between the gene polymorphism and the herpes history.

Other skew sources were the impact of Mexican population admixture, and the linkage disequilibrium (LD). On the one hand, the *F*_*IS*_ found was related to inbreeding more than heterozygous excess, which is one of the main signatures of population stratification [39]. Likewise, the AMOVA test did not exhibit any difference between cases and controls (*p* ≥ 0.05), being the primary variation source within individuals, probably related to the studied men’s birthplaces. To adjust, the birthplace variable, the bias might diminish significantly. In light of this evidence, the birthplace has been considered as a valid proxy for the ancestry markers [40]. Nevertheless, the population stratification correction using ancestry markers is the best strategies to avoid the population stratification bias [41]; consequently, it is likely a residual confusion in our results. However, the association of the risk allele with the ancestral lineages could be a right approach to avoid spurious associations. On the other hand, the LD found between rs10993994 and different polymorphisms on 10.q11 (i.e., rs7071471, rs7081532, rs11006207, rs3123078, rs7075697, rs7077830, rs2611489) could also mean a bias. These genetic markers have not been studied in the Mexican population yet, in turn, is not possible to rule out this confounder factor. However, the studies in Asian and European populations suggest the genetic marker evaluated herein is the strongest related to PC (OR = 1.64; _95%_CI = 1.47 – 1.82), supporting our findings. Hence, our results should be interpreted in light of these limitations.

To our knowledge, this is the first study where the T variant of the rs10993994 polymorphism and its relation to PC was evaluated in Latino populations. Our findings suggest that, under recessive and codominant heritability models, the rs10993994-T allele could contribute to the early-onset of prostate cancer development in the Mexican men. Although the findings found in the present study were consistent with previous reports among different ethnicities, further population-based genetic studies in a more lengthen sample of Mexican men (Native Americans and Mestizos) should be done. These studies should improve the STIs measurement to confirm the risk allele frequency avoiding type II statistical errors. The results found herein could constitute a relevant strategy in further PC analyses, where the onset age ought to be considered. Ultimately, our study could also reinforce the ethnic and even inter-ethnic variation, which was exhibited, indirectly, through the dissimilitude frequencies found between the geographic regions (Central-West *vs* South regions), possible indicative of a genetic susceptibility.

## MATERIALS AND METHODS

### Study subject selection

A population-based case-control study was carried out from November 2011 to August 2014 including unrelated men residents from Mexico City without any record of other types of cancer (age range 42–94 years old). Cases were subjects with incident PC diagnosis identified at six specialty hospitals (four third level hospitals and two second level hospitals) without any restriction regarding their clinical stage. All these cases were histologically confirmed, and based on the Gleason score [30] at diagnosis, these were classified according to its aggressiveness as: well differentiated, ≤ 6; moderately differentiated, = 7; and poorly differentiated, ≥ 8. The individuals whose PC was diagnosed before reaching the age of 60 were designed as early-onset PC, if diagnosis occurred at or after reaching the age of 60, it was considered as late-onset PC. Out of 468 eligible cases, 402 agreed to participate (85.9%). Each case was paired by age (± 5 years) with two healthy men without any PC history, symptomatology associated with probably benign or malignant prostate disease (i.e. hematuria, dysuria, among others) or those who reported a previous study of prostate antigen (PSA) ≥ 4 ng/mL. Using the Master Sampling Frame (National Health Survey), we identified 920 potential controls, and 805 of them agreed to participate (85.5%). For control identification, 33 basic geo-statistical areas (BGAs) were selected. From each BGA in the sample, ten blocks were chosen and finally, starting from the northeast corner of the block, we visited and knocked on each household door to determine whether a male met the eligibility criteria and if there were two or more eligible subjects, one was randomly chosen. Based on those subjects with DNA sample, our final sample size was 322 cases and 628 controls with a ratio Controls/Cases of 1.96. All participants signed an informed consent validated letter by the Ethics Committee of the Mexican National Institute of Public Health (CI: 980); this study was conducted in agreement with the principles established by the Declaration of Helsinki.

### Face-to-face interviews

**Figure 2:**
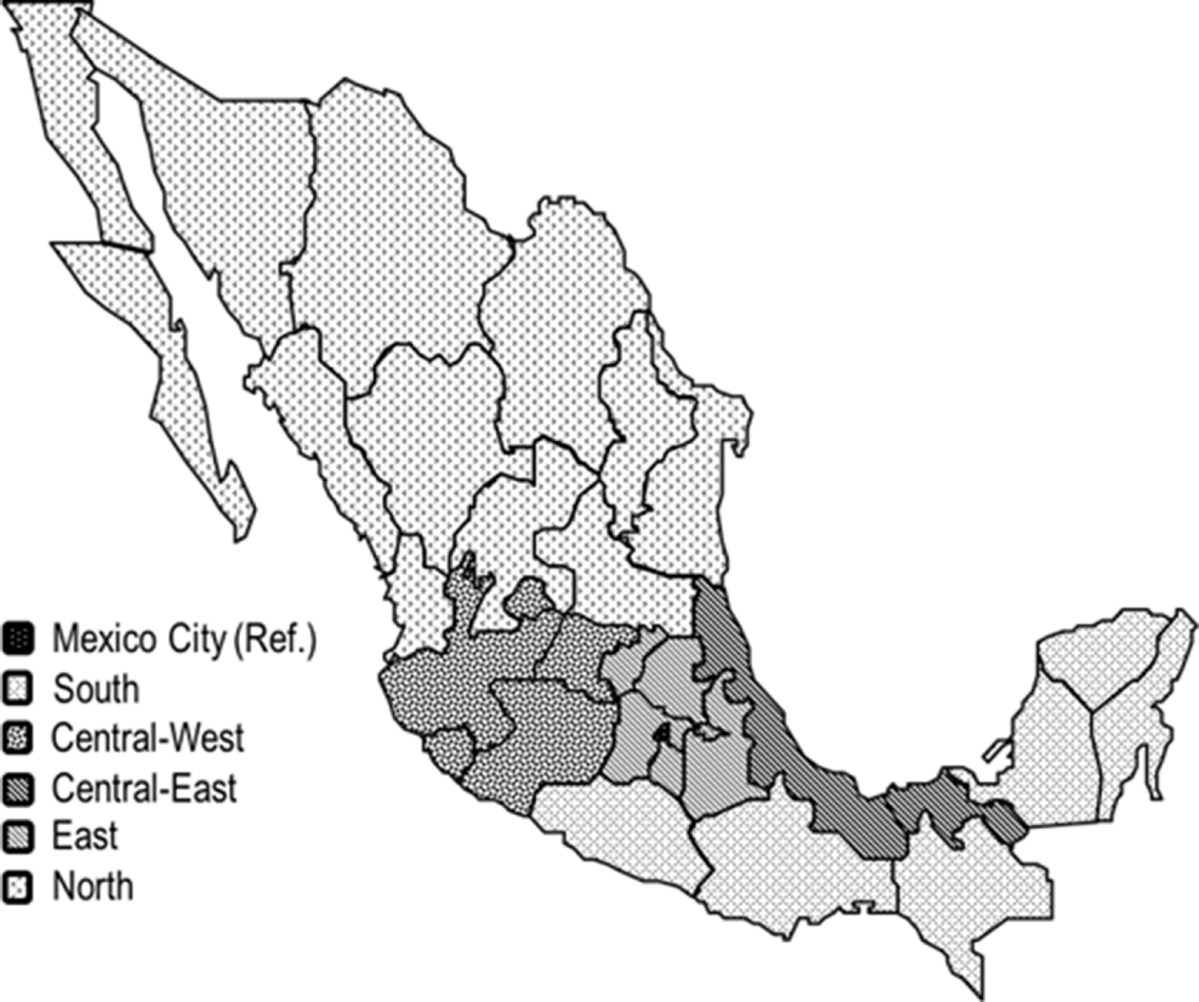
Birthplace according geographic regions Mexico City (Ref.). South: Campeche, Chiapas, Guerrero, Oaxaca, Quintana Roo and Yucatán. Central-West: Aguascalientes, Colima, Guanajuato, Jalisco and Michoacán. Central-East: Hidalgo, Estado de México, Morelos, Puebla, Querétaro and Tlaxcala. North: Chihuahua, Coahuila, Durango, San Luis Potosí, Zacatecas, Baja California, Baja California Sur, Sinaloa, Sonora, Nayarit, Nuevo León and Tamaulipas. East: Veracruz and Tabasco.

Face-to-face interviews were carried out with highly trained staff. Socio-demographic features (i.e., age, place of birth, occupation, and schooling), first-degree PC family history, as well as STIs, physical activity, and smoking histories were determined. Regarding place of birth, six geographic regions were studied (Figure 2). Chronic diseases background (i.e., diabetes type 2, hypercholesterolemia, and hypertension) was also determined in all subjects as presence or absence. Sexual history information included sexual activity age initiation, sexual relationships with sex workers and/or males, and the number of sexual partners that was categorized according to its tertiles distribution between controls. The number of episodes and the start age of STIs such as gonorrhoea, syphilis, genital warts, herpes or chancroid ulcers were also inquired.

Smoking information (age of initiation, frequency and number of cigarettes) was collected for three life’s stages (≤ 20 yr, 21–30 yr, and ≥ 31 yr). If participants did not smoke at the moment of the interview the age when they quit smoking was also obtained. According to the smoking index, calculated for each life stage, two smoking patterns were identified [31]: pattern (A) individuals with low but constant smoking intensity, and pattern (B), individuals whose smoking intensity increased from 30 years old.

A similar procedure was followed regarding the history of physical activity. Through a validated questionnaire we obtained the moderate intensity (≥ 3 METs) and vigorous intensity (≥ 6 METs) of physical activity in three life stages: 15–18 yr, 19–29 yr, and ≥ 30 yr. Three physical activity trajectories throughout life were determined: (A) vigorous physical activity during adolescence, which decreased throughout life; (B) continual low-intensity physical activity throughout life; (C) vigorous physical activity during throughout life.

Anthropometric measurements such as weight (kilograms, kg), height (meters, m), and abdominal circumference (centimetres, cm) were done at interview. Because body mass index (BMI) could has been affected by the disease under study this variable was estimated based on reported weight for two years before diagnosis or interview. Dietary habits surveys in PC cases were obtained taking as time frame the three years previous to diagnosis and three years before the interview for controls.

### Molecular study

Peripheral venous blood (7 mL) was obtained from each participant using the Vacutainer system (Becton Dickinson, Franklin Lakes, NJ, USA); the mononuclear package was compiled using Ficoll-hypaque (Sigma Aldrich, St Lous, MO, USA). DNA was isolated from mononuclear cells with TRIzol (Thermo Fisher Scientific, Suwanee, GA, USA). The purity (λ260/λ280) and concentration were evaluated using Nano-Drop 1000 (Thermo Fisher Scientific, Suwanee, GA, USA); DNA integrity was checked using agarose (Bioline) gels 0.8% stained with ethidium bromide (Sigma Aldrich, St Lous, MO, USA).

Allelic discrimination of the rs10993994 polymorphism was determined using a TaqMan Genotyping assay (Applied Biosystems, Carlsbad, CA, USA) using Viia 7 Real-Time PCR System (Applied Biosystems, Carlsbad, CA, USA) following manufacturer instructions. All experiments were performed in duplicate.

To evaluate the possible contribution of ancestral background in PC development Y-chromosome haplogroups were determined in 49 early-onset PC individuals. The haplotypes were determined by 17 hypervariable markers with Y-Filer kit (Applied Biosystems, Carlsbad, CA, USA); the resulting amplicons were carried out using capillary electrophoresis (ABI 3130XL Genetic Analyzer, Applied Biosystems, Carlsbad, CA, USA). The haplogroups were assigned using a fitness score; the probability of identifying the ancestral lineage was done using the haplogroup predictor software (http://www.hprg.com/hapest5/).

### Statistical analysis

Cases and controls were compared according to selected characteristics depending on the variable, Chi-square or t-Student was used. Allelic and genotypic frequencies of *MSMB* polymorphism, Hardy-Weinberg expectations, and analysis of molecular variance (AMOVA) were obtained with Arlequin v3.5. Multidimensional scaling plots (MDS) were determined using SPSS v11, this analysis included related ancestral populations obtained from 1000 genomes project (http://www.internationalgenome.org/).

The genetic associations (allelic and genotypic) between the *MSMB* polymorphism and PC were done using unconditional logistic regression; the genotypic association was evaluated using dominant (CC *vs* CT+TT), co-dominant (CC *vs* CT and *vs* TT), and recessive (CC+CT *vs* TT) models. Independent models were used to determine the association between risk variant and PC aggressiveness (well, moderated, and poorly-differentiated), as well as between risk variant and start age (early- and late-onset).

Age (at interview) was included as a continuous variable in the bivariate and multivariate models. We evaluated as potential confounders the following features: birthplace, familiar first-degree history of PC, the history of chronic and STIs diseases, smoking habits, BMI two years before diagnosis or interview and physical activity. In the final models for whole PC, only remain: birthplace and the familiar first-degree history of PC; early-onset PC model was also adjusted by BMI. The effect modification between history of STIs and the allelic variants (rs10993994) were evaluated including an interaction term between both variables; *p* ≤ 0.10 was considered as statistically significant. All analyses were carried out using STATA v14 software package (Stata Co, College Station, TX, USA).

## SUPPLEMENTARY MATERIALS TABLES



## Abbreviations

AMOVA: Analysis of molecular variance
BGAs: geo-statistical areas
BMI: Body mass index
CVM: Central Valley of Mexico
HWD: Hardy-Weinberg departure
LD: Linkage disequilibrium
MDS: Multidimensional scaling plots
METs: Metabolic equivalents
PC: Prostate cancer
PSP94: Prostate secretory protein 94 amino acids
STIs: Sexually transmitted infections
SNP: Single nucleotide polymorphism
