# Supplementary material for: Contribution of MSMB promoter region gene polymorphism to early-onset prostate cancer risk in Mexican males
Source: Oncotarget. 2019 Jan 22;10(7):738–48. doi: 10.18632/oncotarget.26592 (PMC6366823; doi:10.18632/oncotarget.26592)
Supplement: Supplementary file 1 [file oncotarget-10-738-s001.pdf]

Contribution of *MSMB* promoter region gene polymorphism to early-onset prostate cancer risk in Mexican males

SUPPLEMENTARY MATERIALS

Supplementary Table 1: Selected characteristics of study population according to allelic distribution of rs10993994 polymorphism among controls

Characteristics	Alele		*p* value[a]
	C<br>*n* = 863	T<br>*n* = 391	
Birthplace[b]			
Mexico City	558 (64.7)	268 (68.5)	0.02
South	69 (8.0)	17 (4.3)	
Central-West	59 (6.8)	39 (10.0)	
Central-East	135 (15.6)	45 (11.5)	
North	20 (2.3)	12 (3.1)	
East	22 (2.6)	10 (2.6)	
Family history of PC			
Yes vs. No	23 (2.7)	11 (2.8)	0.88
History of Chronic Diseases			
Yes vs. No	353 (40.9)	175 (44.6)	0.21
History of STIs			
Yes vs. No	100 (11.6)	42 (10.7)	0.66
History of Gonorrhea			
Yes vs. No	53 (6.1)	21 (5.4)	0.59
Number of sexual partners			
≤ 2	280 (33.0)	134 (34.6)	
3–6	299 (35.2)	139 (35.9)	0.70
> 6	270 (31.8)	114 (29.5)	
Smoking patterns[c]			
No smokers	298 (34.5)	124 (31.6)	
A	504 (58.3)	238 (60.7)	0.61
B	62 (7.2)	30 (7.7)	
Physical activity patterns[d]			
None	93 (10.8)	33 (8.4)	
A	161 (18.6)	71 (18.1)	0.57
B	552 (63.9)	258 (65.8)	
C	58 (6.7)	30 (7.7)	
Body Mass Index[e]			
Normal	228 (27.4)	96 (25.1)	
Overweight	390 (46.9)	206 (53.9)	0.08
Obesity	214 (25.7)	80 (20.9)	

[a]Test Chi-[2].

[b]**Birthplace**: Mexico City (Ref.). South: Campeche, Chiapas, Guerrero, Oaxaca, Quintana Roo and Yucatán. Central-West: Aguascalientes, Colima, Guanajuato, Jalisco and Michoacán. Central-East: Hidalgo, Estado de México, Morelos, Puebla, Querétaro and Tlaxcala. North: Chihuahua, Coahuila, Durango, San Luis Potosí, Zacatecas, Baja California, Baja California Sur, Sinaloa, Sonora, Nayarit, Nuevo León and Tamaulipas. East: Veracruz and Tabasco.

[c]**Smoking patterns**: A: males who reported low and constant smoking intensity, behavioral; B: males with an initial period of low smoking intensity, followed by an increase after 30 years old.

[d]**Physical activity patterns**: A: males who had high PA intensity and frequency at 15–18 years old and showed a higher reduction throughout life; B: males who maintained consistently low PA, and C: males who consistently performed more PA.

[e]**BMI**: two years before interview or diagnosis.

Supplementary Table 2: Association between rs10993994 polymorphism in MSMB gene and total PC according to sexually transmitted infection history

MSMB polymorphism	Sexually Transmitted Infection History							
	No				Yes			
	Cases	Controls	OR[a]	95% CI	Cases	Controls	OR[a]	95% CI
Allele								
C	337	763	1.00	-	105	100	2.58	1.88–3.52
T	147	349	0.98	0.81–1.23	53	42	2.75	1.73–4.38
	p for interaction = 0.82							
Codominant								
CC	120	277	1.00	-	40	37	2.85	1.70–4.77
CT	97	209	1.16	0.83–1.62	25	26	1.97	1.06–3.65
TT	25	70	0.86	0.52–1.45	14	8	4.38	1.58–12.11
	p for interaction = 0.41							
Dominant								
CC	120	277	1.00	-	40	37	2.84	1.70–4.75
CT+TT	122	279	1.08	0.79–1.48	39	34	2.44	1.44–4.12
	p for interaction = 0.56							
Recessive								
CC+CT	217	486	1.00	-	65	63	2.44	1.64–3.62
TT	25	70	0.81	0.49–1.33	14	8	4.37	1.58–12.10
	p for interaction = 0.29							

[a]Model adjusted for age at interview, birthplace and family history of PC.
